# Who Is Suffering from the “Corona Blues”? An Analysis of the Impacts of the COVID-19 Pandemic on Depression and Its Implications for Health Policy

**DOI:** 10.3390/ijerph182312273

**Published:** 2021-11-23

**Authors:** Sunhee Kim, Seoyong Kim

**Affiliations:** 1 Department of Local Government Administration, Gangneung-Wonju National University, Gangneung-si 25457, Korea; shkim7675@hanmail.net; 2 Department of Public Administration, Ajou University, Suwon 16499, Korea

**Keywords:** depression, anxiety, corona blues, COVID-19 pandemic, health policy

## Abstract

COVID-19 is tremendously affecting not only social structures but also people’s psychological states. In particular, COVID-19 is negatively affecting psychological health, in particular, the depression. When individuals are experiencing the depression, there is increase in the suicide rate and occurrence of serious social problems. This study therefore examines factors affecting depression by using hypothesis testing. Previous studies have limitations in that they focus only on demographic variables or other specific variables. In contrast, this study focuses on the influences of four non-pandemic and seven pandemic-related variables on people’s depression. We analyze data from a social survey (N = 1525) in Korea which adopted the stratified quota sampling method. Results show that, first, among the demographic variables, young people experience depression to a greater extent than older people do. Second, among the non-pandemic variables, individuals with more social support, good health, optimism, and self-efficacy exhibit lower levels of depression. Third, among the factors related to COVID-19, fear of infection, financial instability, personal lifestyle changes, and poor health status increase depression.

## 1. Introduction, Theoretical Issues and Hypothesis

### 1.1. Why Do Corona Blues Matter?

Since the World Health Organization (WHO) declared a pandemic on 11 March 2020, 207,173,086 cases of COVID-19, including 4,361,996 deaths, have been confirmed as of 16 August 2021 [1]. COVID-19 has fundamentally changed both the social structure at the macro level and individuals’ ways of life at the micro level. The economy has staggered and unemployment has risen across countries. Owing to COVID-19, individuals have been deprived of the right to move freely in their social lives, and many of them face economic difficulties. In addition, COVID-19 has fundamentally changed the quality of people’s lives. For example, Solomou and Constantinidou [2] found that a large percentage (48%) of respondents in Cyprus reported significant financial concerns, and 66.7% reported significant changes to their quality of life. Moreover, the pandemic has seriously affected not only individuals’ physical health but also their mental health. The data show that rates of anxiety, depression, alcohol use disorder, and lower mental well-being have been higher during the COVID-19 pandemic [3]. According to Wang et al. [4], 16.5% of individuals reported moderate-to-severe depressive symptoms, 28.8% reported moderate-to-severe anxiety symptoms, and 8.1% reported moderate-to-severe stress levels. Lee et al. [5] show that both healthcare workers and severe acute respiratory syndrome (SARS) survivors experienced mental health problems during the SARS epidemic. Additionally, Jeong et al. [6] reported that during the Middle East respiratory syndrome outbreak in 2015, noninfected but quarantined people exhibited anxiety symptoms and feelings of anger. The psychological shocks due to COVID-19 are remarkably large compared to those related to previous pandemics or everyday situations. Bueno-Notivol et al. [7] report that the pooled depression prevalence of 25% in the case of COVID-19 appears to be seven times higher than the estimated global prevalence of depression, which was 3.44% in 2017. Moreover, because the overall global prevalence of anxiety disorders is estimated to be 7.3% in normal times [8], Santabárbara et al. [9] report that anxiety levels in the general population have increased threefold to 25% during the COVID-19 outbreak (95% confidence interval (CI): 21–29%).

Additionally, COVID-19 has had a greater impact on the psychological health of individuals directly involved in COVID-19-related work. For example, Liu et al. [10] analyzed the mental well-being of medical staff working around COVID-19 and found higher rates of depression (50.7%), anxiety (44.7%), insomnia (36.1%), and stress-related symptoms (73.4%). In addition, Chew et al. [11] show that depression has increased among healthcare workers. Moreover, Al Maqbali et al. [12] found that nurses are at a higher risk of distress, anxiety, depression, and sleep disturbances owing to COVID-19. Depression can serve as a barrier to rational medical and mental health interventions during pandemics [4]. In other words, an individual’s mental health problems are highly likely to cause social distrust and lead to the mistrust of public authorities, non-adherence to infection control measures, and the stigmatization of particular groups’ behavior [13].

Although many studies have investigated mental health after COVID-19 among the general public, few studies have systematically analyzed the causes of depression by using a more theoretical background and hypothesis tests. This study aims to analyze the causal factors affecting depression by using hypothesis testing. Because previous studies focus only on demographic variables or other specific variables, they exclude theoretically and practically meaningful variables. Instead, this study considers not only sociodemographic factors but also four non-pandemic variables and seven pandemic-related variables as the predictors of depression.

### 1.2. Theoretical Issues: Impact of COVID-19 on Mental Health

Depression (major depressive disorder) is a common and serious medical illness that negatively affects a person’s feelings, thoughts, and actions [14]. Depression is a normal reaction to a sudden worsening of living circumstances that involves separation and uncertainty [15]. When people are exposed to uncontrollable events, they express helplessness and a lack of motivation, resulting in depression [16].

Because the COVID-19 pandemic is an uncontrollable event, it is surely affecting individuals’ depression. For example, Ni et al. [17] showed that about one-fifth of a set of 1577 respondents in China reported probable depression (N = 303, 19.21%, 95% CI: 17.3–21.2%). Moreover, Ahmed et al. [3] reported that more than one-third of respondents in their study (37.1%) exhibited different forms of depression (mild, 10.2%; moderate, 17.8%; and severe, 9.1%). Moreover, Al Banna et al. [18] examined the effect of the COVID-19 pandemic on mental health for a representative sample of home-quarantined Bangladeshi adults and found that the rates of anxiety and depressive symptoms were 33.7% and 57.9%, respectively.

Moreover, the impact of the COVID-19 pandemic is so great that it is difficult to compare it with previous outbreaks. For example, Chew et al. [11] reported that during previous epidemics (e.g., SARS, H1N1 influenza, and Ebola), rates of anxiety in the general population ranged from 3.2% to 12.6%. In the case of the COVID-19 pandemic, Salari et al. [19] reported that the prevalence of depression in 14 studies with a total sample size of 44,531 people was 33.7% (95% CI: 27.5–40.6%). Bueno-Notivol et al. [7] found a sevenfold increase in the depression rate among the general population of patients with COVID-19.

Rates of depression vary greatly across different empirical studies because these studies examine different subjects. Salari et al. [19] reported different rates of depression ranging from 27.5% to 40.6% among 14 studies. Bueno-Notivol et al. [7] conducted a meta-analysis of 12 studies and found that the prevalence of depression ranged from 7.45% to 48.30%. Noticeably, depression varies widely across countries. Porter et al. [20] reported that the rates of symptoms of at least mild depression were highest in Peru at 32% (95% CI: 29.49–33.74%) and lowest in Vietnam at 9% (95% CI: 8.33–10.77%).

Previous studies of depression have three limitations. First, most previous studies focus on demographic variables. Ahmed et al. [3] analyzed differences in depression according to age, gender, and province, and Kazmi et al. [21] examined the impacts of age, gender, and employment on depression. Second, few studies compare influencing factors; instead, studies focus on the impacts of specific variables on psychological problems during the COVID-19 pandemic. For example, Bäuerle et al. [22] focused on subjective levels of information, Guo et al. [23] focused on social media use, and Huang et al. [24] focused on knowledge. Nguyen et al. [25] studied the effects of changes in health literacy. Third, efforts to verify the effects of factors related to the COVID-19 pandemic on depression have been insufficient. Few studies analyze the influence of pandemic factors on mental health. For example, Porter et al. [20] emphasized the roles of pandemic-related stressors, such as health risks, economic adversity, food insecurity, and educational or employment disruption, all of which are risk factors for anxiety and depression. Moreover, Santabárbara et al. [9] concluded that causal factors for anxiety include not only several sociodemographic variables, such as being female, being younger, marital status, educational level, and social status, but also pandemic-related variables, such as social isolation, financial instability, insufficient knowledge of COVID-19, epidemiological or disease clinical risk, and some lifestyle variables.

Considering these limitations of previous studies, we analyzed the effects of demographic (six variables), pandemic-related (seven variables), and non-pandemic-related factors (four variables) on depression by putting forth 17 hypotheses. We expect that testing these hypotheses will help to build a theory of depression during the COVID-19 pandemic.

### 1.3. Sociodemographic Factors

#### 1.3.1. Gender

The many comparative studies of depression in men and women show that women generally experience depression more than men do [26]. For example, Al Banna et al. [18] found that the prevalence of depressive and anxiety symptoms is significantly higher among women (45.6% and 64.3%, respectively). Fu et al. [27] explained that women have a higher rate of depression than men do because women are socialized to have more emotional experiences than men or have differences in brain chemicals and hormones. However, this explanation has limitations in the context of the COVID-19 pandemic because it considers a longer period of socialization and does not account for the external pandemic situation. Thus, Rodríguez-Rey et al. [28] explained the increased burden on women during the COVID-19 pandemic from a labor perspective. Women are mainly responsible for caregiving work at home, which has been directly affected by the lockdowns due to COVID-19.

Since COVID-19 has critically influenced the depression [29], a few studies report different findings. Wang et al. [4] reported increased anxiety scores in men during the COVID-19 pandemic. Their explanation is that men may have high anxiety because they perform many outdoor activities that can be affected by COVID-19. Moreover, Ahmed et al. [3] found no significant differences in depression and other measures of mental well-being across men and women.

**Hypothesis 1 (H1)**. *Women exhibit more depression than men do during the COVID-19 pandemic*.

#### 1.3.2. Age

In the context of the COVID-19 pandemic, younger people have higher levels of depression than older people do. According to the Corona 19 National Mental Health Survey [30] conducted in Korea, in the first quarter of 2021, the proportions of people in their 20s and 30s at risk for depression were 30.0% and 30.5%, respectively, more than twice as high as the proportion of those in their 60s (14.4%). After examining the effects of the COVID-19 pandemic in China, Ahmed et al. [3] reported that people aged 21–40 were in a more vulnerable position in terms of their mental health and well-being. Salari et al. [19] showed that during the pandemic, levels of anxiety, depression, and stress were significantly higher among people aged 21–40. Moreover, some studies show that even within groups of younger people, the youngest members are more exposed to depression. Al Banna et al. [18] found that higher rates of depressive symptoms are associated with being younger (≤23 years) (62.8%). Mental illness rates differ by age group. For example, Kazmi et al. [21] found that depression is high among respondents between 15 and 35 years old, whereas anxiety is prevalent among those aged 21–25. Santabárbara et al. [9] explain that younger people are more likely to face uncertainties about their jobs and futures, experience financial instability, and obtain information from social media. These factors fuel depression among young people and negatively affect mental health.

**Hypothesis 2 (H2)**. *Younger people are more likely to experience depression than older people are*.

#### 1.3.3. Education Level

Generally, educational level is negatively related to psychological illness [28]. Solomou et al. [2] and Lei et al. [29] showed that lower education levels are associated with higher anxiety and depression scores during the COVID-19 pandemic. In addition, Salari et al. [19] reported that people with higher levels of education have greater levels of anxiety, depression, and stress. Guo et al. [23] analyzed the effects of education levels on depression in the context of COVID-19 and showed that the prevalence of depression is lower among individuals with a college education (OR = 0.69, 95% CI: 0.53–0.91) or a master’s degree (OR = 0.46, 95% CI: 0.63–0.85) than among those with a middle school education. Similarly, Wang et al. [4] reported that during the COVID-19 pandemic, uneducated status is significantly related to higher depression scores.

However, some studies find the opposite result. In a study in China, Zhang and Ma [31] showed that people with higher levels of education have a higher prevalence of symptoms of mental illness owing to a greater awareness of their own health.

**Hypothesis 3 (H3)**. *Individuals with lower educational levels are likely to have higher levels of depression relative to individuals with higher educational levels*.

#### 1.3.4. Income

High incomes can act as a shield against psychological distress during pandemics [32]. Lei et al. [31] found that the highest average household income group in their study (>9000 yuan) had a significantly lower level of depression than the lowest average household income groups (<1500 and 1500–3000 yuan). In addition, according to Shevlin et al. [33], in the United Kingdom (UK), low-income status is a predictor of depression symptoms.

Low social status [25] and economic losses [29] can contribute to higher rates of depression. After the COVID-19 outbreak, widespread economic damages have caused many people to experience psychological issues [3]. Lei et al. [29] showed that after the COVID-19 pandemic, those who did not experience economic losses had significantly lower levels of depression than those in other groups did. These findings suggest that lower incomes increase individuals’ depression.

**Hypothesis 4 (H4)**. *Individuals with low incomes experience more depression than those with high incomes do*.

#### 1.3.5. Marital Status

Marital status systematically influences individuals’ objective quality of life and subjective mental health. Families formed through marriage can create psychological stability because the members provide social support to each other. According to Shah et al. [34], the presence of family leads to lower levels of stress, anxiety, and depression. Lei et al. [29] showed that during the COVID-19 pandemic, those who were divorced or widowed had significantly more anxiety and depression than those with other marital statuses had, and those who were single had significantly more depression than those who were married or cohabiting had. Similarly, Shah et al. [34] reported that single and divorced people experience depression. Moreover, Sigdel et al. [35] found that individuals living alone are significantly more likely to have depression.

However, married people may also be highly depressed because they worry about not only their own health but also that of their families. Fu et al. [27] demonstrated that married people exhibit poorer psychological health than unmarried people do.

**Hypothesis 5 (H5)**. *Married people are less depressed than unmarried, divorced, or widowed people are*.

#### 1.3.6. Children

Generally, children bring happiness to their parents. However, the risks that children face in the COVID-19 pandemic are likely to place a burden on parents. In particular, childcare for school-aged children places a burden on parents when schools are closed because of the pandemic. Many studies show that depression increases when people have children or when the number of children in a family increases. Shevlin et al. [33] showed that in the UK, the presence of children at home predicts the criteria for either anxiety or depression and trauma symptoms. Conversely, during the COVID-19 pandemic, depression and anxiety were reduced among those with fewer than two children who are 10 years of age or older and not living together [26]. These findings imply that anxiety and depression increase when a household includes many infants and young children with a high parenting burden.

**Hypothesis 6 (H6)**. *People with more children are more likely to feel depressed than those with fewer children are*.

### 1.4. Non-Pandemic Factors

#### 1.4.1. Social Support

Several studies report that weak social support is strongly correlated with poor psychological well-being during the pandemic [36,37,38,39,40]. In the case of the COVID-19 pandemic, greater social support leads to lower rates of depression [9], whereas lower levels of social support can contribute to higher rates of depression [17]. Such social support is associated with a lower likelihood of depression not only for people in general but also among professionals [17]. In contrast, Lei et al. [29] demonstrated that having no psychological support is significantly associated with higher depression scores.

Depression linked to the specific context of the COVID-19 pandemic may be best addressed with supportive interventions [7]. During a pandemic, public support rather than personal social support can help to relieve depression. According to Lei et al. [29], among individuals who were unaffected by COVID-19, those who did not receive psychological support or counseling from the community or government agencies were more likely to have higher depression scores.

**Hypothesis 7 (H7)**. *People with stronger social support are less depressed than those with weaker social support are*.

#### 1.4.2. Health Status

Health status is closely associated with depression. Shevlin et al. [33] argue that depression symptoms are influenced by an individual’s health and the health of others. According to Lei et al. [29] and Guo et al. [23], those with “very good” self-perceived health exhibit lower levels of depression than those in other groups do. Moreover, Wang et al. [4] find that poor self-rated health status is significantly associated not only with a greater psychological impact of the pandemic but also with higher levels of depression (*p* < 0.05). A personal history of chronic illness [4,33] or medical problems [39] increases depression rates. According to Wang et al. [4], chills, myalgia, cough, dizziness, coryza, and sore throat were significantly associated with higher levels of depression during the COVID-19 pandemic. Moreover, psychological illness is high among those living with a chronically ill patient [23].

**Hypothesis 8 (H8)**. *People in good health are more likely to have lower levels of depression than those in poor health are*.

#### 1.4.3. Optimism

Based on a review of 175 studies published from 1980 to 2007, Kotov et al. [41] found that mental disorders are strongly linked to personalities and have similar trait profiles. Among personality types, neuroticism, agreeableness, and conscientiousness are positively related to intense psychological illnesses, such as distress [32]. Mazza et al. [39] found that personality traits also influence depression rates during the COVID-19 pandemic; those who score higher on negative affect and detachment report higher rates of depression. Among various personality traits, this study focuses on optimism. Optimism may significantly affect mental and physical well-being by promoting a healthy lifestyle and adaptive behaviors and cognitive responses. It is associated with greater flexibility, higher problem-solving capacity, and the more efficient elaboration of negative information ([42], p. 25). Zhou et al. [43] found that optimistic and positive thoughts and attitudes toward the COVID-19 pandemic help to reduce depression. In addition, Biber et al. [44] showed that optimism is negatively associated with general anxiety disorder during the COVID-19 pandemic.

**Hypothesis 9 (H9)**. *Individuals who are more optimistic have lower levels of depression relative to those who are not optimistic*.

#### 1.4.4. Self-Efficacy

Self-efficacy is a kind of degree of feeling about locus of control [45]. Generally, high self-efficacy is associated with low levels of depression [41]. In the context of the COVID-19 pandemic, Mumtaz et al. [46] showed that self-efficacy not only has a direct negative effect on depression but also moderates the relationship between the fear of COVID-19 and depression. Arima et al. [47] found that self-efficacy and self-esteem are both influential predictors of psychological distress during the COVID-19 pandemic. Both self-efficacy and its associations with specific behaviors help to reduce depression. For example, Bressington et al. [48] showed that respondents reporting self-efficacy to wear masks properly are less likely to report depression.

In the context of the COVID-19 outbreak, formal interventions to increase self-efficacy are needed to reduce depression. Sun et al. [49] showed that individuals who receive psychiatric training have higher positive emotions and self-efficacy and that providing psychological assistance online increased self-efficacy.

**Hypothesis 10 (H10)**. *People with high self-efficacy have lower levels of depression than those with low self-efficacy have*.

### 1.5. Pandemic Factors

#### 1.5.1. Concerns about Infection and Threats to One’s Life

Exposure to the coronavirus and high estimates of personal risk of COVID-19 infection can affect anxiety, depression, or trauma symptoms [33]. Hyland et al. [50] show that depression is associated with COVID-19 infection and a higher perceived risk of COVID-19 infection. This finding suggests that risk perception or concerns about infection may be more important for depression than actual infections are. Lei et al. [29] demonstrated that those with greater worry about being infected have higher depression scores. According to Choi et al. [51], concerns about infection are linked to both depression and anxiety.

The perception of the risk of infection for family members and acquaintances may also affect mental health. Wang et al. [4] showed that high levels of worry about other family members with COVID-19 are significantly associated with higher stress subscale scores. In addition, individuals with friends or relatives who contracted COVID-19 reported higher levels of depression and anxiety [28,39].

**Hypothesis 11 (H11)**. *Greater concerns about infection and threats to one’s life increase depression*.

#### 1.5.2. Financial Instability

COVID-19 has had negative effects on individuals’ financial situations. Financial crises are highly likely to lead to an increase in depression. Empirical studies show that a decrease in income or financial pressure [52] or a loss of income due to COVID-19 [33] are significantly associated with depressive symptoms. In the context of the COVID-19 pandemic, individuals’ financial situations deteriorate over time. Lei et al. [29] demonstrate that greater property damage is significantly associated with higher depression and anxiety scores. In Japan, Ueda et al. [53] showed that respondents who felt that they were financially worse off relative to a year ago had higher depressive symptoms than those whose financial situations had not deteriorated. Additionally, some studies reported that a high income can help prevent psychological illness during the pandemic [32,54,55].

**Hypothesis 12 (H12)**. *Individuals whose personal financial situations worsened owing to COVID-19 are more depressed than those whose personal financial situations did not worsen*.

#### 1.5.3. Employment Instability

Work status can influence not only individuals’ economic stability but also their psychosocial conditions through a lack of daily routines and scheduling [21]. Socioeconomic factors, particularly employment status, can contribute to depression rates. Individuals who are unable to return to their original jobs owing to unemployment or quarantine will face very high levels of psychological illness. Those who perceive a high risk of job loss caused by COVID-19 also express higher levels of psychological illness [33,50]. In addition, during the COVID-19 pandemic, characteristics of depression have been more visible among unemployed individuals than among employed individuals [18,21,34]. Both unemployment status and job stability can influence depression. According to Ueda et al. [53], part-time and temporary contract-based workers are more likely to suffer from depressive symptoms.

**Hypothesis 13 (H13)**. *People with high levels of occupational instability, particularly those who are unemployed, have higher levels of depression than those with more occupational stability have*.

#### 1.5.4. Lifestyle Changes

A history of stressful situations is associated with higher levels of depression and anxiety [39]. Owing to the COVID-19 pandemic, people’s lifestyles have been greatly changing. In extreme cases of mandatory quarantine, people ceased social interactions and became isolated. Such lifestyle changes affect people’s mental health. For example, when people experience forced isolation, their mental health is negatively affected by reduced social interactions and changes in routines [23]. Hawryluck et al. [56] examined quarantines during SARS outbreaks and their psychological effects and reported that the effects of quarantines on mental health increased as their durations increased. Similarly, Shah et al. [34] reported that during the quarantines and lockdowns for COVID-19, 50.9% of participants exhibited aspects of anxiety, 57.4% showed signs of stress, and 58.6% exhibited depression. In addition, Lei et al. [29] compared depression rates among people who were affected and unaffected by quarantines during the COVID-19 outbreak in China in early February 2020. They showed that the former group had higher rates of depression than the latter group did. Shah et al. [34] reported that a large number of days spent in quarantine and a lack of exercise caused by COVID-19 were associated with increased stress, anxiety, and depression.

However, Rodríguez-Rey et al. [28] reported different results, finding that people who are more physically active or engaged in hobbies have lower depression and anxiety levels.

**Hypothesis 14 (H14)**. *Individuals with increased lifestyle changes (i.e., more isolated personal lives) due to COVID-19, especially those with individual-centered isolated life patterns, will have a higher degree of depression relative to those with no such changes*.

#### 1.5.5. Health Status Changes after COVID-19 Infections

It is natural for anxiety to increase when health conditions worsen [57]. Those who experienced the threat of death from COVID-19 have higher psychological responses, such as anxiety [10,18]. Nguyen et al. [25] show that in Vietnam, those who contracted COVID-19 had a higher likelihood of depression. COVID-19 directly affects the health of the elderly. Seong et al. [58] showed that the deterioration of health and the increase in loneliness among the elderly due to COVID-19 are positively correlated with depression. Confirmed and suspected COVID-19 patients have higher rates of anxiety (63.90%) and depression (55.40%) relative to other cohorts [10]. These studies show that worsening health status after COVID-19 increases individuals’ depression.

**Hypothesis 15 (H15)**. *People whose health deteriorates after COVID-19 have higher depression levels than those whose health does not deteriorate*.

#### 1.5.6. Knowledge

The COVID-19 pandemic creates substantial uncertainty, which is linked to anxiety and depression. Because uncertainty about the future is a function of information and knowledge, increases in information and knowledge reduce uncertainty. Thus, obtaining sufficient and timely information during a crisis, such as the COVID-19 pandemic, helps with psychological adaptation. Regular updates with the latest information about COVID-19 are related to lower levels of depression [22]. Wang et al. [4] showed that additional information on the availability and effectiveness of medicines and vaccines lowers depression. Thus, individuals who spend more time accessing information about COVID-19 are significantly more likely to have lower depression levels [35]. Moreover, Huang and Zhao [24] demonstrate that knowledge of COVID-19 is negatively associated with depressive symptoms.

**Hypothesis 16 (H16)**. *Those with more knowledge about COVID-19 have lower levels of depression than those with little knowledge have*.

#### 1.5.7. Preventive Actions

Since the COVID-19 outbreak, individuals have adopted protective actions, such as wearing masks and social distancing. Governments have encouraged citizens to take preventative actions. Such protective actions affect not only individuals’ objective health statuses but also their subjective mental health. For example, Aldhmadi et al. [59] found a negative association between depression scores and preventive actions, such as frequent hand washing, wearing a mask when going out, social distancing, and covering the nose when sneezing. Moreover, causal analysis showed that adhering to precautionary measures is a significant predictor of lower depressive symptoms. A variety of equipment is required for protective actions, and a lack of such equipment can cause depression. Sakib et al. [60] showed that healthcare professionals who used the same protective equipment for a week had greater depression levels than others did. Additionally, Bressington et al. [48] showed that respondents reporting higher reuse of masks and the use of masks for self-protection are more likely to report being depressed.

**Hypothesis 17 (H17)**. *People who engage in preventive behaviors have lower levels of depression relative to those who do not do so*.

## 2. Sample and Measurements

### 2.1. Sample

This study analyzes survey data collected from the public in Korea. We designed the questionnaire and Korea Research, an opinion-polling agency, distributed the questionnaire and collected responses from 6 August 2020 to 11 August 2020. The study population includes men and women over the age of 18 from all over Korea and the sampling frame is the Korea Research Access Panel. This panel consists of 460,000 people nationwide and the survey was conducted through the web (the URL was sent via text messages and e-mails from mobile phones). In total, 1525 people completed all of the questions. The sample was drawn to be representative of Koreans. The characteristics of the population are based on the results of a census survey conducted in 2020. We conducted quota sampling to ensure the representativeness of the sample and the gender and age ratios of the sample therefore almost coincide with the characteristics of the general population in Korea. In our sample, 16.7% of respondents are 18–29 years old (N = 254), 16.3% are 30–39 years old (N = 248), 19.6% are 40–49 years old (N = 299), 20.3% are 50–59 years old (N = 310), and 27.1% are over 60 years old (N = 414). In terms of gender, 47.9% of respondents are male (N = 720) and 52.1% are female (N = 805). The maximum allowable sampling error in this study is ±2.5% at the 95% confidence level, assuming random sampling.

### 2.2. Measurements

We designed our measurements after examining previous surveys on the psychological impacts of pandemics, such as SARS and influenza outbreaks [61]. In addition, we refer to questions offered by official agencies on COVID-19. For example, the WHO [62] provides a survey tool and guidance for monitoring knowledge, risk perception, preventive behaviors, and trust to inform the response to the pandemic. In addition, we referred to COVID-19 survey questionnaires conducted in specific countries. Since the outbreak of COVID-19, several countries have officially executed surveys to monitor people’s behavior. For example, the Australian Bureau of Statistics (ABS) has carried out the Household Impacts of COVID-19 Survey several times to obtain information about the prevalence and nature of the impacts of COVID-19 on households in Australia (see https://www.abs.gov.au accessed on 1 July 2020) [63]. This survey asks Australians about feelings that affect their emotional and mental well-being and are associated with experiences of anxiety and depression. It also asks people how frequently in the previous four weeks they felt nervous, so nervous that nothing could calm them down, hopeless, restless or fidgety, so sad that nothing could cheer them up, and worthless. After excluding the measurements of anxiety and including those of depression, we adopted four items from the ABS: “hopelessness,” “sadness,” “worthlessness,” and “depression.” These four items are frequently adopted by other depression measures, such as the Beck Depression Inventory (BDI), originally created by Beck et al. [64] and revised by Beck et al. [65]. In the BDI, hopelessness measures pessimism, sadness measures mood, and worthlessness measures the sense of failure. We asked respondents to rate how often they felt these four depression symptoms after the COVID-19 outbreak on a five-point scale (1 = very rarely, 5 = very often). The reliability level for the four items (Cronbach’s alpha) is 0.939.

Table 1 contains descriptions of the measurement items except for depression. We chose our survey questions so that the constructs are as similar as possible to those used in previous studies [61,63]. For all measurement items except concerns about infection and threats to one’s life, lifestyle changes, and preventive actions, we presented a specific statement to respondents and asked them whether they agreed with it (1 = strongly disagree, 5 = strongly agree). We combined multiple measurement items into one item using average values.

The reason the subject of this study is related to COVID-19 is that when the measurement items of the dependent variable and the independent variable are composed, we include the contents related to COVID-19. When measuring depression in the dependent variable, the question is “How was your status for the following items (“hopelessness”, “sadness”, “worthlessness”, and “depression”) after the outbreak of the corona crisis?” The question includes the issues of COVID-19. Furthermore, the content of COVID-19 is included in the independent variables such as self-efficacy, worry about infection and life-threating, financial instability, occupational instability, lifestyle changes, health status changes, and knowledge. The inclusion of COVID-19 in measurements provides the reasons for making sense of the paper title.

### 2.3. Analysis Method

For statistical analysis in this study, frequency analysis, mean analysis, correlation analysis, and regression analysis were performed. In the frequency analysis, we analyzed responses to four questions measuring depression. In the mean analysis, we examined how the average values of depression vary according to demographic variables. For correlation analysis, simple correlation analysis and partial correlation analysis were performed. The partial correlation analysis controls for gender, age, education level, income, marital status, and number of children. We perform regression analyses with depression as the dependent variable and with pandemic or non-pandemic factor (seven variables) as independent variables. We used SPSS as the statistical analysis package. Normality verification was determined by calculating kurtosis and skewness. As for the dependent variable depression, kurtosis was 0.071 and skewness was 0.894, which was smaller than the reference values of 4 and 2, respectively.

## 3. Analysis

### 3.1. Descriptive Analysis

**Figure 1:**
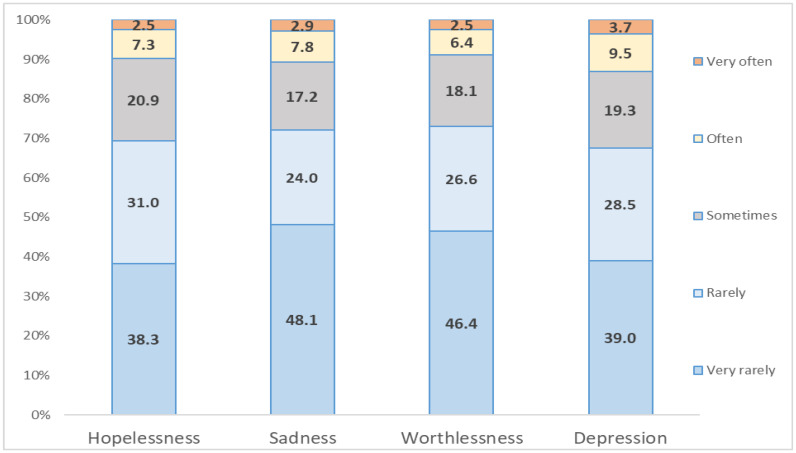
Frequency analysis.

We analyzed the frequencies of the responses to our four depression measures (hopelessness, sadness, worthlessness, and depression). Figure 1 shows the results. Overall, the most frequent response among the five response categories is “very rarely,” followed by “rarely,” “sometimes,” “often,” and “very often.” Notably, this ordering is the same across the four depression measures. This result indicates that people’s responses to the depression measurement items are consistent. The means of the four responses are 2.05, 1.93, 1.92, and 2.10 out of five points for hopelessness, sadness, worthlessness, and depression, respectively. Thus, depression is slightly higher than the other measures. The variances of hopelessness, sadness, worthlessness, and depression are 1.106, 1.221, 1.121, and 1.285, respectively. They indicate that reported levels of depression differ more across respondents.

We divided the respondents into groups according to their sociodemographic characteristics and mean values. If a respondent’s value for a specific variable is lower than the average for all respondents, then that respondent is placed in the low group, and if a respondent’s value is high, then that respondent is placed in the high group. We analyzed differences in depression across different groups, where depression is defined as the average of the four measurement items.

**Figure 2:**
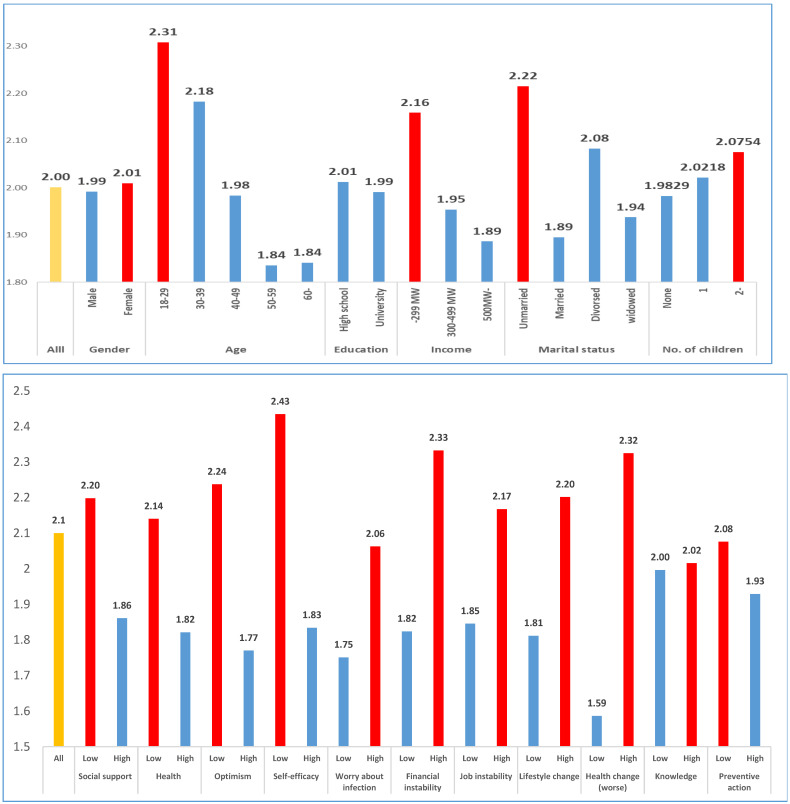
Mean levels of depression for high and low groups.

In Figure 2, first, in terms of gender, previous studies confirm that women consistently exhibit higher depression levels than men do. However, in this study, the difference between men and women is not statistically significant (F-value = 0.114, *p*-value = 0.736). Next, in terms of age group, lower ages are associated with higher levels of depression. In particular, respondents between the ages of 18 and 29 exhibit high levels of depression. Conversely, depression is relatively low among respondents age 50 and older. The difference between the young and old groups is statistically significant (F-value = 13.238, *p*-value = 0.000). In terms of education level, respondents with relatively low educational attainment (i.e., below high school) have higher levels of depression than respondents with a college education or higher have. However, the difference is not large, nor is it statistically significant (F-value = 0.176, *p*-value =0.675). Regarding income, depression differs greatly across the high- and low-income groups; the latter group has a higher level of depression than the former group does. The difference between the two groups is statistically significant (F = 9.917, *p*-value = 0.000). This difference indicates that people with lower incomes are more likely to suffer because of COVID-19, which leads to depression. Regarding marital status, depression levels are higher among unmarried than among married respondents. However, divorced and widowed respondents, who were previously married, also showed high levels of depression. This difference is statistically significant (F-value = 11.003, *p*-value = 0.000). Additionally, the presence of children affects the degree of depression. Depression levels are greater among respondents with large numbers of children. Often, school shutdowns due to COVID-19 cause the childcare system to collapse or function poorly, ultimately increasing the burden on parents. This burden of parenting is likely linked to depression. However, the difference between the two groups is not statistically significant (F-value = 0.779, *p*-value = 0.459).

Respondents receiving less social support have higher levels of depression than those receiving more social support have (F-value = 43.373, *p*-value = 0.000). In the context of the COVID-19 pandemic, social support based on relationships is an intangible asset that provides psychological stability. Regarding health status, depression levels are higher among respondents in poor health than among those in good health, and this difference is statistically significant (F-value = 39.072., *p*-value = 0.000). In the context of the COVID-19 pandemic, poor health plays a very important role in individuals’ qualities of life, as it can increase the likelihood of a COVID-19 infection. Depression is higher among less optimistic respondents than among more optimistic respondents (F-value = 88.039, *p*-value = 0.000). This result suggests that although depression is influenced by objective conditions, such as individuals’ material assets, resources, and physical health, individuals’ psychological attitudes and temperaments also play an important role. Finally, depression is higher in the low self-efficacy group than in the high self-efficacy group. Because COVID-19 can be perceived as uncontrollable by an individual, a sense of efficacy plays an important role in that it allows individuals to have a sense of control (F-value = 119.075, *p*-value = 0.000).

The group with greater concerns about COVID-19 infection and the threat to their lives has higher levels of depression than the group with lower levels of concern has (F-value = 22.532, *p*-value = 0.000). These results suggest that both actual COVID-19 infections and the fear of such infections affect depression. As a result, it is important to provide people with a sense of security that they may not be infected with COVID-19. For financial and occupational instability, the groups with lower levels of stability exhibit higher levels of depression than the groups with higher levels of stability do. Both instability measures have statistically significant impacts (financial instability: F-value = 95.145, *p*-value = 0.000; job instability: F-value = 40.547, *p*-value = 0.000). If financial and job uncertainty are increasing owing to COVID-19, these factors affect individuals’ feelings of depression.

Individuals’ lifestyles changed significantly when the COVID-19 pandemic hit. This study measures lifestyle changes by the amounts of time individuals spent using their smartphones, watching TV, exercising, and developing themselves. All of these activities are related to more individualized uses of time. Depression is higher among respondents who spend more time on personal-oriented activities than among respondents who spend less time on these activities. This difference is statistically significant (F-value = 60.622, *p*-value = 0.000). Interestingly, this result implies that exercise can negatively affect an individual’s psychological state. Depression levels are higher in the group with worsened health status after COVID-19 than in the group whose health did not worsen (F-value = 236.079, *p*-value = 0.000). This result suggests that both health status and changes in health status play important roles.

We measure knowledge by asking respondents how much they know about COVID-19. The results show that people with more knowledge of COVID-19 have greater feelings of depression than those with less knowledge do. However, this difference is not statistically significant (F-value = 0.107, *p*-value = 0.744). Although knowledge usually performs the function of resolving uncertainty, these results show that in the case of COVID-19, this function does not operate well. Lastly, the group that performs more preventive actions has a higher level of depression than the group that does not perform as many such actions (F-value = 8.238, *p*-value = 0.004). These results suggest that preventive measures that are mainly promoted by the government paradoxically play a significant role in reducing individuals’ psychological health.

We perform simple and partial correlation analyses of the relationships between depression and other variables. In Table 2, the cells below the diagonal show the coefficients of the simple correlation analysis, and the cells above the diagonal show the partial correlation coefficients. The partial correlation analysis controls for gender, age, education level, income, marital status, and number of children.

First, looking at the results of the simple correlation analysis, we observe that depression has a positive relationship with concerns about infections and threats to one’s life, financial instability, occupational instability, and decline in health status. It has a negative relationship with social support, health status, optimism, and preventive actions. The variable with the largest correlation with depression is change in health status (0.420). This result may arise because depression and health status are similar attributes; depression is a type of mental health. In addition, depression is highly correlated with financial instability, fear of infection and threats to one’s life, and optimism. Health status after COVID-19, financial instability, and concerns about infection and threats to one’s life are all directly related to COVID-19, suggesting that COVID-19 can directly increase depression among individuals.

The values and significance levels of the partial correlation coefficients are similar to those of the simple correlation coefficients. These results suggest that variables besides demographic variables are consistently associated with depression.

### 3.2. Regression Analysis

We perform regression analyses with depression as the dependent variable, with the results shown in Table 3. Before performing this analysis, we checked the prerequisite conditions for regression analysis. We find no multicollinearity because the tolerance limit (tolerance) is 0.1 or higher and the variance expansion factor value is less than 10 for all variables. The Durbin–Watson value is 2.009, which is between the reference values of one and three, indicating that there is no residual independence problem. The reference groups for the dummy variables are male for gender, less than high school graduation for education level, below five million won for income, and unmarried for marital status.

Age is the only statistically significant demographic variable. Feelings of depression decrease when people grow older. Gender and marital status, which affect depression according to many previous studies, do not have a statistically significant influence on depression in this study. In particular, the fact that all demographic variables except age have statistically insignificant effects suggests that factors besides social demographics play an important role in determining depression in the COVID-19 pandemic.

Next, among the non-pandemic variables, which are not directly related to COVID-19, social support, health status, optimism, and self-efficacy have negative effects on depression. Based on the standardized regression coefficient values, optimism appears to be the most influential variable among the four non-pandemic-related predictors in reducing depression. Optimism plays a more important role than social support and health status do, suggesting that maintaining a positive mindset is more important for avoiding depression than objective physical health or support resources are.

Among the seven variables directly related to the COVID-19 pandemic, concerns about infection and threats to one’s life, financial instability, lifestyle changes, and decline in health status positively affect depression, whereas preventive actions negatively affect depression. Based on the standardized regression coefficient values, health status after COVID-19 has the greatest impact on depression among these variables, followed by lifestyle changes and financial instability. As the influence of these three pandemic-related variables is greater than that of the non-pandemic-related variables, we can observe that COVID-19 situation plays an important role in the increase in depression after COVID-19.

Interestingly, employment instability does not affect depression. This finding may result from the fact that many of the respondents did not have jobs. Knowledge also has no statistically significant effect, suggesting that knowing more about COVID-19 may randomly increase or decrease depression.

The explanatory power of the model, indicated by the adjusted R^2^ value, is 36.1%. Because this value is not very high, it is necessary to identify additional variables that can influence depression. We perform separate regression analyses of each of the three factors to examine the predictive power of each factor. When only the demographic variables are used as independent variables, the adjusted R^2^ value is 4.1%. The four non-pandemic factors have an adjusted R^2^ value of 13.1%. Finally, the seven pandemic-related factors have an adjusted R^2^ value of 29.5%. These results suggest that depression among the public is closely related to the specific COVID-19 context.

## 4. Discussion and Implications

The purpose of this study was to analyze which variables affect the extent of depression in the context of the COVID-19 pandemic. In particular, this study attempted to compare the effects of sociodemographic, non-pandemic, and pandemic-related factors. The main findings can be summarized as follows:

Among the six demographic variables, age is the only variable with a significant influence on depression. We find that depression increases with age. Gender and marital status, which are typically found to affect depression, do not have a significant effect on depression in our analysis. These results suggest that factors other than social demographics play an important role in depression during the COVID-19 pandemic. Next, the variables that are not directly related to COVID-19, that is, social support, health status, optimism, and self-efficacy, affect depression negatively. In particular, optimism plays a major role in reducing depression relative to other variables. Finally, concerns about infection and threats to one’s life due to COVID-19, financial instability, lifestyle changes, and reductions in health status after contracting COVID-19 increase depression whereas preventive actions reduce depression.

Overall, factors related to the COVID-19 pandemic have more significant effects on depression than the demographic factors and non-pandemic-related factors do. The variable that has the greatest impact on depression is change in health status after COVID-19 (worse), followed by lifestyle changes and financial instability.

The practical implications of this study can be summarized as follows. First, this study identifies which groups are vulnerable to depression. These results can be reflected in public health policy by promoting measures to relieve depression among vulnerable groups. Ahmed et al. [3] suggest various programs to help overcome depression and other mental health issues during the COVID-19 pandemic, such as restrictions on media exposure, treatment and training plans, online counseling platforms, guaranteed care for vulnerable people, and rehabilitation programs. Moreover, our study shows that variables related to COVID-19 have greater impacts on depression than demographic or other variables do. This result suggests that it is necessary to manage depression among the public by considering situational factors related to COVID-19.

In terms of health policy, it is necessary to consider the findings of our study to reduce depression. Our findings show that concerns about infection and threats to one’s life influence depression. “Worry” is a kind of risk perception that is formed through both formal communication channels and social media. Media channels instill the fear of COVID-19 in the public. Thus, to lower people’s perception of the risk of infection, it is necessary to control fake news in the media. A strategy is needed to provide health information that can induce the perception of safety.

Next, our findings show that financial instability increases depression. To reduce financial instability, it is necessary to strengthen financial services, such as emergency funds or disaster support, to provide people with a financial safety net.

This study shows that the rise in isolated lifestyles after COVID-19 has been shown to increase depression. Huang and Zhao [66] argue that spending too much time thinking about the outbreak is harmful to one’s mental health. To reduce the negative impact of isolation, it is necessary to prepare communication channels or public health programs to promote interactions between individuals. Wang et al. [4] suggest that the use of electronic devices and applications to provide counseling can decrease the psychological problems caused by COVID-19 and, thus, can induce social stability.

Our findings also demonstrate the critical role of individual health status after a COVID-19 infection. Thus, healthcare programs should be strengthened to manage people’s health better. For example, health management information can be provided, and health checkups can be strengthened at the community level. Because preventive actions reduce depression, it is desirable for the government to encourage people to take preventive actions to respond to COVID-19 actively. It is necessary to publicize the fact actively that such preventive actions can reduce depression in addition to their primary effects of stopping infections.

To bolster social support, it is necessary to strengthen the interactions between individuals within the local community. This support can not only be carried out at the individual level but should also be carried out by formal health agencies at the public health level. Public health authorities can send personal e-mails asking how people are doing or can strengthen their interactions through online meetings. Because self-efficacy and optimism can reduce depression, it is necessary to enable individuals to enhance these traits. We recommend running mindfulness or psychological reinforcement programs to instill positive psychology and a sense of control in individuals.

## 5. Conclusions

This study analyzed the variables that are influencing depression among the public during the COVID-19 pandemic. We found that social support, health status, optimism, self-efficacy, and preventive actions decrease depression, whereas concerns about infection and threats to one’s life, financial instability, lifestyle changes toward individual activities, and worsening health status after COVID-19 increase depression.

This study provides theoretical contributions because previous studies have not systematically examined the effects of factors related to the COVID-19 pandemic on depression. In future study, more verification is needed for the effects of some variables, such as concerns about contagion, financial instability, individual-centric lifestyle changes, and preventative actions, which significantly affect depression according to the results of this study. In addition, it is necessary to verify whether and why employment instability and knowledge have no significant effects. Moreover, further research is needed to determine why demographic variables other than age have no effect on depression.

This study demonstrates that factors related to COVID-19 affect depression. However, it has some limitations. First, we focused on seven variables related to the COVID-19 pandemic, but other important variables may exist. There are many factors that influence depression. Generally, gender, age, and educational background have an impact on demographics. Also, not only personal or social factors such as social relationships and domestic violence, but also physical and genetic factors influence depression. Among sociodemographic variables, Ciarambino et al. [67] reported that older, less educated female relatives were likely to have an unsatisfactory perception of the way the COVID-19 case was handled. Moreover, Ciarambino et al. [68] detected that the infection is more frequent in males, and the number of male patients hospitalized in ICU (Intensive Care Unit) is also higher than females. Related to personal factors, Biegler [69] pointed out that personal autonomy provides a strong additional reason to recommend psychotherapy in depression. In the case of social relationships, Sherwood et al. [70] demonstrated that care recipients’ mental status and recency of care demands had a near significant indirect effect on elderly caregiver’s depressive symptoms. Furthermore, Sahlini and Tushar [71] showed that the present review reveals that, similar to previous pandemics and epidemics, there has been an alarming rise in the incidents of gender-based violence during the COVID-19 pandemic. Moreover, regarding physical and genetic factors influencing depression, Seney et al. [72] showed that sex chromosome complement regulates expression of mood-related genes. Pařízek et al. [73] demonstrated that the strongest correlations between steroid and postpartum depression change. Other variables other than demographic variables were not included in this study, which is a limitation of this paper.

Second, this study falls short of a full measurement in that we used very simple four words to measure depression. Future studies should use more comprehensive measures of depression. Third, because this study was conducted at one particular time during the COVID-19 pandemic, its cross-sectional design causes some limitations. It will be useful to measure depression in different contexts. Thus, longitudinal studies on depression over time are needed. Fourth, because this study uses Internet-based surveys, the results may be biased if some people could not be contacted.

## Figures and Tables

**Table 1:** Concepts, measurement items, and the scale’s reliability.

Concept	Measurement	Scale	Mean (S.D.)	Cronbach’s α
Social support	-I have many good social relationships with people. -When I am in trouble, I can get help from others.	Five-point scale (1 = strongly disagree, 5 = strongly agree).	3.49 (0.690)	0.800
Health status	-I am healthy. -I am in good health compared to other people.	Five-point scale (1 = strongly disagree, 5 = strongly agree).	3.24 (0.808)	0.901
Optimism	-I am an optimist who thinks the future will be bright. -I think many of the current problems will be resolved.	Five-point scale (1 = strongly disagree, 5 = strongly agree).	3.33 (0.771)	0.828
Self-efficacy	-If I make an effort, I can fully practice preventive actions. -I have sufficient ability to practice coronavirus prevention actions.	Five-point scale (1 = strongly, 5 = strongly agree).	3.95 (0.719)	0.868
Worry about infection and life-threating	-How do you rate the probability of being infected with the coronavirus on a scale from 1 to 10, where 1 point means “very low’ and 10 points means “very high” -How do you rate the probability of your life being threatened by the coronavirus on a scale from 1 to 10, where 1 point means “very low” and 10 points means “very high”	Ten-point scale (1 = very low, 10 = very high)?	4.30 (2.188)	0.833
Financial instability	-What do you think about the impact of COVID-19? I will personally go bankrupt.	Five-point scale (1 = strongly disagree, 5 = strongly agree).	3.08 (1.108)	-
Occupational instability	- I may be personally out of work or lose my job.	Five-point scale (1 = strongly disagree, 5 = strongly agree).	3.36 (1.129)	-
Lifestyle changes	-How much did your participation in the following activities increase compared to before the outbreak of the coronavirus: (1) time spent using a smartphone, (2) time spent watching TV, (3) time exercising, and (4) time for self-development? Responses: ① decreased, ② the same, ③ slightly increased, ④ increased a lot.	Four-point scale (1 = decreased. 2 = no change, 3= increased a little, 4 = increased a lot)	2.64 (0.610)	0.628
Health status changes	-Physical health deteriorated after the COVID-19 pandemic. -Health become worse after the COVID-19 pandemic.	Five-point scale (1 = strongly disagree, 5 = strongly agree).	2.78 (0.840)	0.771
Knowledge	-I am well aware of the COVID-19 pandemic. -I know more about the COVID-19 pandemic.	Five-point scale (1 = strongly disagree, 5 = strongly agree).	3.02 (0.650)	0.840
Preventive actions	-Have you taken the following preventive actions to prevent coronavirus infection: (1) covering your mouth with your sleeve when coughing, (2) ventilating the room at least twice a day, and (3) wearing a mask?	Five-point scale (1 = did not comply at all, 5 = thoroughly complied)	4.17 (0.635)	0.769

**Table 2:** Pearson simple and partial correlations.

	1	2	3	4	5	6	7	8	9	10	11	12
**1. Depression**		−0.16 **	−0.217 **	−0.289 **	−0.229 **	0.306 **	0.318 **	0.239 **	0.251 **	0.43 **	−0.003	−0.125 **
**2. Social support**	−0.164 **		0.254 **	0.335 **	0.267 **	−0.097 **	−0.056 *	−0.024	0.069 **	−0.038	0.257 **	0.299 **
**3. Health status**	−0.219 **	0.290 **		0.337 **	0.197 **	−0.262 **	−0.1 **	−0.083 **	0.074 **	−0.15 **	0.185 **	0.171 **
**4. Optimism**	−0.311 **	0.346 **	0.353 **		0.243 **	−0.165 **	−0.129 **	−0.116 **	0.037	−0.117 **	0.152 **	0.15 **
**5. Self−efficacy**	−0.239 **	0.274 **	0.210 **	0.257 **		−0.178 **	−0.06 *	0.06 *	−0.01	−0.177 **	0.135 **	0.433 **
**6. Concern about infection and threat to life**	0.309 **	−0.104 **	−0.265 **	−0.169 **	−0.175 **		0.202 **	0.149 **	0.131 **	0.296 **	0.008	−0.06 *
**7. Financial instability**	0.326 **	−0.097 **	−0.131 **	−0.147 **	−0.066 **	0.216 **		0.741 **	0.121 **	0.262 **	0.054 *	0.015
**8. Occupational instability**	0.260 **	−0.070 **	−0.119 **	−0.145 **	0.044 †	0.165 **	0.753 **		0.100 **	0.235 **	0.017	0.106 **
**9. Lifestyle change**	0.253 **	0.076 **	0.079 **	0.029	−0.01	0.129 **	0.111 **	0.093 **		0.163 **	0.093 **	0.043 †
**10. Health status change**	0.420 **	−0.041	−0.146 **	−0.110 **	−0.170 **	0.296 **	0.265 **	0.239 **	0.160 **		0.08 **	−0.033
**11. Knowledge**	−0.013	0.275 **	0.202 **	0.175**	0.142 **	0.005	0.032	−0.010	0.090 **	0.077 **		0.161 **
**12. Preventive actions**	−0.131 **	0.292 **	0.176 **	0.146 **	0.439 **	−0.046 †	0.018	0.098 **	0.045 †	−0.018	0.152 **	

Note: † *p* < 0.05; * *p* < 0.01; ** *p* < 0.001.

**Table 3:** Regression analysis.

Factor	Variable	B	S.E.	Beta	T-Value	Sig
	Constant	1.902	0.232		8.194	0.000
Factor 1: Sociodemographic factors	Gender	−0.036	0.043	−0.018	−0.839	0.401
	Age	−0.007	0.002	−0.111	−3.332	0.001
	Education level	−0.003	0.045	−0.002	−0.077	0.939
	Income	−0.021	0.048	−0.009	−0.433	0.665
	Married	−0.095	0.071	−0.046	−1.344	0.179
	Divorced	−0.038	0.111	−0.008	−0.341	0.733
	Widowed	−0.029	0.157	−0.004	−0.188	0.851
	Number of children	0.017	0.032	0.013	0.530	0.596
Factor 2: Non-pandemic factors	Social support	−0.064	0.034	−0.045	−1.864	0.062
	Health status	−0.072	0.029	−0.059	−2.483	0.013
	Optimism	−0.220	0.031	−0.171	−7.205	0.000
	Self-efficacy	−0.090	0.034	−0.065	−2.688	0.007
Factor 3: Pandemic related factors	Concern about infection and threats to life	0.046	0.010	0.101	4.474	0.000
	Financial instability	0.154	0.029	0.172	5.366	0.000
	Employment instability	−0.002	0.028	−0.002	−0.074	0.941
	Lifestyle changes	0.296	0.035	0.182	8.541	0.000
	Health changes (worse)	0.327	0.027	0.276	12.142	0.000
	Knowledge	0.016	0.034	0.010	0.467	0.641
	Preventive actions	−0.071	0.038	−0.045	−1.857	0.064
F-value	46.203 ***					
R^2^/adjusted R^2^	0.369/0.361					
F1: R^2^/adjusted R^2^	0.041/0.036					
F2: R^2^/adjusted R^2^	0.131/0.133					
F3: R^2^/adjusted R^2^	0.295/0.272					

Note: *** *p* < 0.001.

## Data Availability

The data presented in this study are available on request from the corresponding author.
